# Vaccination with Tetanus, Diphtheria, and Acellular Pertussis Vaccine of Pregnant Women Enrolled in Medicaid — Michigan, 2011–2013

**Published:** 2014-09-26

**Authors:** Michelle Housey, Fan Zhang, Corinne Miller, Sarah Lyon-Callo, Jevon McFadden, Erika Garcia, Rachel Potter

**Affiliations:** 1 Michigan Department of Community Health; 2 CDC/Council of State and Territorial Epidemiologists Applied Epidemiology Fellowship; 3 Immunization Services Division, National Center for Immunizations and Respiratory Diseases, CDC; 4 Career Epidemiology Field Officer Program, Division of State and Local Readiness, Office of Public Health Preparedness and Response, CDC

In October 2011, the Advisory Committee on Immunization Practices (ACIP) first recommended the routine administration of a tetanus, diphtheria, and acellular pertussis vaccine (Tdap) during pregnancy as a strategy to protect infants from pertussis (also known as whooping cough) (1). This recommendation applied to women previously unvaccinated with Tdap and specified the optimal vaccination time as late second or third trimester (after 20 weeks’ gestation) (1). By vaccinating pregnant women, infants, who are at highest risk for mortality and morbidity from pertussis, gain passive immunity from maternal antibodies transferred to them in utero (2–4). Since this recommendation was made, little has been published on the percentage of women receiving Tdap during pregnancy. In Michigan, Medicaid pays for costs of pregnancy for approximately 40% of births (5). Infants enrolled in Medicaid are a particularly vulnerable population; in Michigan, their all-cause mortality is higher than that of privately insured infants.* To assess vaccination coverage among pregnant women enrolled in a publicly funded insurance program in Michigan, Medicaid administrative claims data and statewide immunization information system data for mothers of infants born during November 2011–February 2013 were analyzed. This report describes the results of that analysis, which indicated that only 14.3% of these women received Tdap during pregnancy, with rates highest (17.6%) among non-Hispanic, non-Arab whites and lowest (6.8%) among Arab women.† Vaccination was related to maternal age and gestational age at birth, but not to adequacy of prenatal care. In 2013, recognizing the importance of Tdap for every pregnancy, ACIP revised its guidelines to include a Tdap dose during every pregnancy (6). Ensuring that all infants receive the protection against pertussis afforded by maternal vaccination will require enhanced efforts to vaccinate pregnant women.

Birth certificates for infants born during November 2011–February 2013 were linked to maternal Medicaid claims data. Only women who delivered their first infant at age ≥18 years and who received full Medicaid benefits for at least 1 month between 20 weeks gestation and live birth were included. Preterm and full-term infants (defined as those delivered at ≤42 weeks’ gestation) were included. Pregnant adolescents were excluded because they should be vaccinated with Tdap based on their age (i.e., 11–18 years), independent of their pregnancy status. Using each infant’s birth date and gestational age at birth, an approximate date of conception was calculated. *International Classification of Diseases, Ninth Revision* (ICD-9) procedural codes for vaccination with Tdap (code 99.39) and the *Current Procedural Terminology* codes for vaccination with Tdap (code 90715) were used to identify vaccinations given any time during pregnancy. Statewide immunization information system data from the Michigan Care Improvement Registry supplemented Medicaid data to capture more complete vaccination histories during pregnancy.

The percentage of women who received a Tdap vaccination at any time during pregnancy and disparities in vaccination administration based on maternal race/ethnicity were evaluated. Maternal race/ethnicity was categorized as non-Hispanic and non-Arab white (“white”), non-Hispanic and non-Arab black (“black”), non-Hispanic and non-Arab Asian (“Asian”), non-Hispanic and non-Arab Native American (“Native American”), Hispanic, or Arab based on the birth certificate. Other potential predictors of vaccination were assessed including gestational age at live birth, plurality of the pregnancy, maternal age at live birth, and adequacy of prenatal care use using the Kotelchuck index (7). Relative risks assessed differences by maternal race and ethnicity adjusting for two significant predictors of vaccination, maternal age and gestational age at delivery. An alpha level of <0.05 denoted statistical significance.

A total of 15,181 women were included in the study (Table 1). The majority of women were white (59.3%), and the second largest racial/ethnic group was black (29.6%). Approximately 65.5% of infants were born full-term (at ≥39 weeks), and the overwhelming majority of pregnancies were singleton (98.7%). Based on the Kotelchuck index, over half of the study population received either intermediate (37.6%) or inadequate (37.5%) prenatal care. The median maternal age at delivery for the entire study population was 21.0 years (Table 1).

Among the study population, 14.3% of women received Tdap during pregnancy (Table 1). Differences in vaccination coverage by maternal race and ethnicity were noted; 17.6% of whites, 8.4% of blacks, 11.9% of Asians, and 21.9% of Native Americans received Tdap during pregnancy. Among Hispanic women, 15.3% received Tdap, whereas 6.8% of Arab women received the vaccine during pregnancy. Based on bivariate analyses, infant’s gestational age (full-term versus pre-term) and maternal age at delivery were significant predictors of Tdap vaccination (p≤0.001). Adequacy of prenatal care was not a predictor of Tdap vaccination. Women whose care was rated “adequate” or “adequate plus” were not more likely to have been vaccinated than women whose care was rated “intermediate” or “inadequate.”

Unadjusted and adjusted relative risks (RRs) and 95% confidence intervals (CIs) for Tdap vaccination were calculated based on maternal race and ethnicity (Table 2). Whites were significantly more likely to receive Tdap compared with blacks (RR = 2.1; CI = 1.8–2.3), Asians (RR = 1.5; CI = 1.1–2.0), and Arabs (RR = 2.6; CI = 1.9–3.7). No significant difference in Tdap coverage was observed between white women and Hispanic women (RR = 1.1, CI = 1.0–1.4) or between white women and Native American women (RR = 0.8, CI = 0.5–1.2).

## Discussion

Based on Medicaid administrative claims data and the statewide immunization information system records, 14.3% of publicly insured women who delivered their first child during November 2011–February 2013 received Tdap during pregnancy. Because the 2011 ACIP recommendation was only for unvaccinated women and women could have received Tdap before pregnancy, a 100% coverage rate for Tdap during pregnancy would not be expected. However, based on data from the 2012 National Health Interview Survey, only 14.2% of adults reported receiving Tdap in the past 7 years (8). With such a low proportion of the general population having received Tdap, a higher proportion of pregnant women in this population would be expected to have received Tdap if ACIP recommendations had been consistently followed.

Black, Asian, and Arab women were significantly less likely to receive Tdap during pregnancy compared with white women, even after controlling for significant predictors of vaccination (infant’s gestational age and maternal age at delivery). No significant difference in vaccination was observed between Hispanic women or Native American women and white women. Racial disparities in prenatal vaccination have also been observed with the influenza vaccination; black women (45.4%) were less likely to receive the influenza vaccine compared with white women (52.2%) (9).

A previous study examining Tdap coverage among privately insured women of reproductive age (regardless of pregnancy status) found that 45.5% of women received Tdap during their lifetime (10). No racial or ethnic differences in receipt of Tdap were observed; almost 80% of the study population were white women (10). Results based on the privately insured population of reproductive age differ from these results for the publicly insured Medicaid population, for whom Tdap coverage was assessed during pregnancy.

The findings in this report are subject to at least six limitations. First, only vaccines administered during pregnancy were captured in the dataset. This study did not capture pre- or post-partum vaccinations because Tdap administrations during those periods are unlikely to provide passive immunity to the infant. Second, the Medicaid administrative claims database only captures vaccinations that were correctly billed to and paid by Medicaid. Third, vaccinations administered at locations other than physicians’ offices might not be included in this dataset. Fourth, because birth records were linked to maternal Medicaid claims to identify a cohort of women, the study population only included women delivering a live infant and cannot represent all pregnant women. Fifth, although no significant difference in vaccination was observed between white women and Native American women, the result should be interpreted with caution because of small sample sizes. Finally, results from this study are based on a publicly insured population and might not be generalizable to other insured populations.

Vaccinating pregnant women remains the best strategy for protecting newborns against pertussis. Effective February 2013, ACIP revised its previous recommendation to include a Tdap dose during every pregnancy, regardless of previous Tdap vaccination history (6). Future studies should reevaluate vaccination coverage to determine whether coverage improved after the 2013 ACIP recommendation. Further exploration into reasons for racial and ethnic disparities in Tdap vaccination also is needed.

What is already known on this topic?

In 2011, the Advisory Committee on Immunization Practices first recommended routine vaccination with tetanus, diphtheria, and acellular pertussis vaccine (Tdap) of pregnant women who had never received it. Vaccinating pregnant women is an important strategy for providing passive immunity to infants, who are at highest risk from pertussis.

What is added by this report?

To assess whether pregnant women enrolled in Medicaid in Michigan were being vaccinated with Tdap during pregnancy, Michigan Medicaid administrative claims data and statewide immunization information system data were analyzed. The analysis indicated that only 14.3% of women received Tdap during pregnancy, with rates highest among non-Hispanic, non-Arab whites and lowest among Arab women. Vaccination was related to maternal age and gestational age at birth, but not to adequacy of prenatal care.

What are the implications for public health practice?

Ensuring that all infants receive the protection against pertussis afforded by maternal vaccination will require enhanced efforts, such as increased education of clinicians, parents, and families.

Increased education for clinicians, parents, and families might increase adherence to ACIP recommendations. Public health programs should encourage the use of immunization registries and immunization prompts, as well as develop better partnerships with clinicians responsible for prenatal vaccinations.

## Figures and Tables

**TABLE 1:** Demographic characteristics and Tdap vaccination status during pregnancy among women aged ≥18 years enrolled in Michigan Medicaid and delivering their first child during November 2011–February 2013

	Overall		Tdap during pregnancy		No Tdap during pregnancy	
			
Characteristic	No.	(%)	No.	(%)	No.	(%)
**Total study population**	**15,181**	**(100)**	**2,168**	**(14.3)**	**13,013**	**(85.7)**
**Maternal race and ethnicity** *						
Non-Hispanic and non-Arab white	8,975	(59.3)	1,583	(17.6)	7,392	(82.4)
Non-Hispanic and non-Arab black	4,477	(29.6)	375	(8.4)	4,102	(91.6)
Non-Hispanic and non-Arab Asian	311	(2.1)	37	(11.9)	274	(88.1)
Non-Hispanic and non-Arab Native American	73	(0.5)	16	(21.9)	57	(78.1)
Hispanic	806	(5.3)	123	(15.3)	683	(84.7)
Arab	484	(3.2)	33	(6.8)	451	(93.2)
**Gestational age at birth**						
<39 weeks	5,235	(34.5)	657	(12.6)	4,578	(87.4)
≥39 weeks (full term)	9,946	(65.5)	1511	(15.2)	8,435	(84.8)
**Plurality**						
Singleton	14,985	(98.7)	2,139	(14.3)	12,846	(85.7)
Multiple	195	(1.3)	29	(14.9)	166	(85.1)
**Kotelchuck index**						
Adequate plus	2,267	(15.7)	322	(14.2)	1,945	(85.8)
Adequate	1,326	(9.2)	171	(12.9)	1,155	(87.1)
Intermediate	5,427	(37.6)	821	(15.1)	4,606	(84.9)
Inadequate	5,413	(37.5)	785	(14.5)	4,628	(85.5)
**Median maternal age at delivery (yrs)**	21		22		21	

**Abbreviation:** Tdap = tetanus toxoid, reduced diphtheria toxoid, and acellular pertussis vaccine.

* Missing values for race and ethnicity (n = 55), plurality (n = 1), and Kotelchuck index (n = 748).

**TABLE 2:** Unadjusted and adjusted relative risks for Tdap vaccination during pregnancy, by maternal race/ethnicity, among women aged ≥18 years enrolled in Michigan Medicaid and delivering their first infant during November 2011–February 2013

	Unadjusted		Adjusted	
		
Maternal race/ethnicity	RR	(95% CI)	RR	(95% CI)*
White† versus black†	2.1	(1.9–2.3)§	2.1	(1.8–2.3)§
White† versus Asian†	1.5	(1.1–2.0)§	1.5	(1.1–2.0)§
White† versus Native American†	0.8	(0.5–1.2)	0.8	(0.5–1.2)
White† versus Hispanic	1.2	(1.0–1.4)	1.1	(1.0–1.4)
White† versus Arab	2.6	(1.9–3.6)§	2.6	(1.9–3.7)§

**Abbreviations:** Tdap = tetanus toxoid, reduced diphtheria toxoid, and acellular pertussis vaccine; RR = relative risk; CI = confidence interval.

* Adjusted for maternal age at delivery and gestational age at birth.

† Non-Hispanic and non-Arab.

§ Statistically significant.

## Footnotes

* Michigan ranks second in the United States for largest Arab population. The Division for Vital Records and Health Statistics at the Michigan Department of Community Health collects Arab ethnicity on the birth certificate.

† Information available at https://michigan.gov/documents/mdch/Infant_Mortality_Final_Deliverable_433533_7.pdf.
